# Entanglement Degradation in Two Interacting Qubits Coupled to Dephasing Environments

**DOI:** 10.3390/e25101458

**Published:** 2023-10-17

**Authors:** Rahma Abdelmagid, Khadija Alshehhi, Gehad Sadiek

**Affiliations:** 1 Department of Applied Physics and Astronomy, University of Sharjah, Sharjah 27272, United Arab Emirates; u22104927@sharjah.ac.ae (R.A.); u17103635@sharjah.ac.ae (K.A.); 2 Department of Physics, Ain Shams University, Cairo 11566, Egypt

**Keywords:** quantum decoherence, open quantum systems, quantum information

## Abstract

One of the main obstacles toward building efficient quantum computing systems is decoherence, where the inevitable interaction between the qubits and the surrounding environment leads to a vanishing entanglement. We consider a system of two interacting asymmetric two-level atoms (qubits) in the presence of pure and correlated dephasing environments. We study the dynamics of entanglement while varying the interaction strength between the two qubits, their relative frequencies, and their coupling strength to the environment starting from different initial states of practical interest. The impact of the asymmetry of the two qubits, reflected in their different frequencies and coupling strengths to the environment, varies significantly depending on the initial state of the system and its degree of anisotropy. For an initial disentangled, or a Werner, state, as the difference between the frequencies increases, the entanglement decay rate increases, with more persistence at the higher degrees of anisotropy in the former state. However, for an initial anti-correlated Bell state, the entanglement decays more rapidly in the symmetric case compared with the asymmetric one. The difference in the coupling strengths of the two qubits to the pure (uncorrelated) dephasing environment leads to higher entanglement decay in the different initial state cases, though the rate varies depending on the degree of anisotropy and the initial state. Interestingly, the correlated dephasing environment, within a certain range, was found to enhance the entanglement dynamics starting from certain initial states, such as the disentangled, anti-correlated Bell, and Werner, whereas it exhibits a decaying effect in other cases such as the initial correlated Bell state.

## 1. Introduction

Quantum information science aims to harness the unique properties of quantum systems for advanced computation, communication, and simulation [1]. However, quantum systems, such as qubits, are highly sensitive to the inevitable interactions with their environment, leading to decoherence and loss of quantum entanglement [2]. In particular, dephasing, which refers to the loss of relative phase information between quantum states, presents a significant challenge for realizing robust quantum technologies. Understanding the behavior of interacting qubits in the presence of dephasing is crucial for mitigating these effects and advancing the quantum information processing (QIP) field. The controllable coherent coupling between qubits is mandatory for enabling powerful quantum computations. However, dephasing can disrupt this coupling and cause the loss of entanglement, hindering the performance of quantum gates and introducing errors in quantum computations [3]. The impact of dephasing is influenced by factors such as the qubit–qubit interaction strength, the specific form of the interaction, and the dephasing mechanism itself. The investigation of the interplay between dephasing and interacting qubits to understand how dephasing affects entanglement dynamics, gate operations, and the overall performance of quantum systems has been in the focus of research in the QIP field. Mitigating the effects of dephasing is a key objective in quantum information science. Researchers have explored various strategies to suppress dephasing-induced decoherence in interacting qubit systems. Dynamical decoupling techniques, such as spin echo sequences and dynamical decoupling pulses, aim to manipulate the qubits’ interactions with the environment to reduce the impact of dephasing [4,5,6]. Another approach is the use of quantum error correction codes [7,8], which redundantly encode quantum information to protect it against errors and decoherence. Additionally, the concept of decoherence-free subspaces allows for encoding information in a subspace that is insensitive to specific forms of dephasing [9,10]. These mitigation strategies seek to enhance coherence, extend qubit lifetimes, and improve the overall reliability of quantum computations in the presence of dephasing.

Several theoretical and experimental works were devoted to studying and exploring the behavior of interacting qubits coupled to dissipative and dephasing environments, especially in systems that are promising candidates for implementing quantum computation and simulation such as superconducting circuits, trapped ions, and semiconductor-based qubits [11,12,13,14,15,16,17,18,19]. In particular, there has been a special interest in studying systems of two qubits coupled to dissipative and dephasing environments, where in most of these works, the two qubits are considered to be identical, while coupled to a single or two separate independent environments (such as the optical, thermal, dephasing, or dissipative) [20,21,22,23,24,25,26,27,28,29,30,31]. In a pioneering work by Yu and Eberly, it was shown that the bipartite entanglement between two originally entangled qubits, which are isolated from each other while coupled to quantum or classical noise, may vanish within a finite time, which they called entanglement sudden death (ESD) [32,33]. In a very relevant work, the entanglement between two interacting identical qubits coupled to separate dephasing environments, starting in a mixed entangled state, was found to exhibit periods of sudden death and rebirth (dark and bright periods) before vanishing completely. The time it takes to entirely vanish was found to be longer than the time needed for the entanglement sudden death in a system of two non-interacting qubits [34]. The entanglement dynamics in a system of two qubits initiated in an extended Werner-like state under the effect of a dephasing channel was studied [35,36]. It was shown that the purity of the initial state significantly affects the entanglement robustness in the noisy channel. The time evolution of a system of two qubits coupled to a classical dephasing environment starting from different initial states and driven by a Gaussian stochastic process was investigated, where it was demonstrated that the engineering of the environment has a very small effect on the sudden death of the entanglement, though it may significantly preserve the entanglement for a long time [37]. The quantum correlation between two independent qubits coupled to classical dephasing environments (singly or collectively) was studied using the local quantum uncertainty (LQU) as a measure [38]. The dynamics of LQU versus that of the entanglement, represented in terms of the concurrence, were considered. It was shown that, while the entanglement exhibits a sudden death, the LQU decays asymptotically. Very recently, it was shown how the uncorrelated pure dephasing of one component of a hybrid system can impact the dephasing rate of the transition in light–matter systems [19].

In this paper, we study a system of two interacting two-level non-identical atoms (qubits) in the presence of pure (uncorrelated) and correlated dephasing environments. We investigate how the asymmetry of the two-qubit system, attributed to their different frequencies and coupling strengths to the environment, affects the entanglement dynamics and asymptotic behavior. We show that the impact of this asymmetry varies significantly depending on the initial state of the system and the degree of anisotropy of their mutual interaction. For certain initial states, the difference in the qubits’ energy gaps (frequencies) may cause higher decay rates and entanglement sudden death, while for others, it could provide an enhancing effect. Furthermore, we demonstrate how the difference in the coupling strength of the uncorrelated dephasing environment generally harms the entanglement and causes rapid decay with a rate that varies depending on the degree of anisotropy and the initial state type. Finally, we present the effect of the coupling to the correlated dephasing environment and show how it may enhance, for a short period of time, or damage the system entanglement depending on its value, the initial state, and the anisotropy of the system.

In fact, several platforms are relevant to our study, for instance, spin qubits, such as electron or nuclear spins in quantum dots, are susceptible to dephasing due to interactions with nearby nuclei, electrons, and other environmental factors. This dephasing can lead to the loss of quantum information stored in the spin states [39,40,41,42]. Furthermore, flux qubits, which are superconducting qubits, are sensitive to magnetic flux changes. They can experience dephasing due to fluctuations in the magnetic environment, leading to the loss of coherence in the qubit states [43,44,45,46]. Moreover, qubits coupled to resonant microwave cavities can experience dephasing due to fluctuations in the cavity modes. This dephasing can impact the fidelity of two-qubit gates and overall quantum circuit performance [47,48,49,50]. Besides, nuclear magnetic resonance (NMR) qubits, which are based on the manipulation of nuclear spins, may dephase due to interactions with other nuclear spins, leading to transverse relaxation, which reduces the coherence time of the qubits [51,52].

This paper is organized as follows: In the next section, we present our model and the solution. In Section 3, we discuss the entanglement dynamics and asymptotic behavior, starting from different initial states, based on our model. We conclude in Section 4.

## 2. The Model and Solution

We considered a system of two interacting asymmetric (non-identical) atoms (qubits), each one characterized by two levels: a ground state and an excited state labeled as $|g_{i}\rangle$ and $|e_{i}\rangle$, where i=1,2 refers to the first and second atoms, respectively. The Hamiltonian of the system is given by

(1) $$H=\omega_{1}S_{1}^{z}+\omega_{2}S_{2}^{z}+J\left(\frac{\left(1+\gamma \right)}{2}S_{1}^{x}S_{2}^{x}+\frac{\left(1-\gamma \right)}{2}S_{1}^{y}S_{2}^{y}+\delta S_{1}^{z}S_{2}^{z}\right),$$

The first two terms in the Hamiltonian represent the asymmetry of the two non-interacting atoms with $\omega_{1}$ and $\omega_{2}$ accounting for the transition frequency of each atom, while the final three terms describe the atom–atom interactions. The spin operator *S* is defined by $S_{i}^{z}=1/2\left(|g_{i}\rangle \langle g_{i}|-|e_{i}\rangle \langle e_{i}|\right)$ and $S_{i}^{+}=|e_{i}\rangle \langle g_{i}|=S_{i}^{x}+iS_{i}^{y}={S_{i}^{-}}^{†}$. Clearly, these operators are monomorphic to the spin 1/2 operators; therefore, we can describe all our system characteristics using the spin system terminology. The parameter *J* represents the atom–atom interaction strength, while the anisotropy parameters γ and δ specify the different types of systems that we may consider: Ising (γ=1 and δ=0), XYZ (γ=0.5 and δ=1), and XXX (γ=1 and δ=0.5). Throughout this paper, we set the parameters ℏ=J=1 for convenience.

We studied the time evolution of the system starting from either an initial pure or a mixed state. Starting with the entangled atoms in a pure state, the wave function of the composite system can be defined, at t=0, as

(2) $$\left|\psi \left(0\right)\rangle =a\;\right|e_{1},e_{2}\rangle +b\;|e_{1},g_{2}\rangle +c\;|g_{1},e_{2}\rangle +d\;|g_{1},g_{2}\rangle ,$$

which is a linear combination of all possible product states of the two atoms and a,b,c, and *d* are arbitrary complex quantities that satisfy the normalization condition:

(3) $${\left|a\right|}^{2}+{\left|b\right|}^{2}+{\left|c\right|}^{2}+{\left|d\right|}^{2}=1.$$

In this basis, the Hamiltonian has the form:

(4) $$H=\frac{1}{4}(\begin{array}{cccc} \delta +2\left(\omega_{1}+\omega_{2}\right) & 0 & 0 & \gamma \\ 0 & -\delta +2\left(\omega_{1}-\omega_{2}\right) & 1 & 0\\ 0 & 1 & -\delta -2\left(\omega_{1}-\omega_{2}\right) & 0\\ \gamma & 0 & 0 & \delta -2\left(\omega_{1}+\omega_{2}\right) \end{array}),$$

On the other hand, the density matrix corresponding to an initial Werner mixed state is given by

(5) $$\rho \left(0\right)=\frac{1}{3}(a|e_{1},e_{2}\rangle \langle e_{1},e_{2}|+d\;|g_{1},g_{2}\rangle \langle g_{1},g_{2}|+\left(b+c\right)|\psi \rangle \langle \psi |),$$

where the wavefunction takes the following form:

(6) $$|\psi \rangle =\frac{1}{\sqrt{b+c}}(\sqrt{b}|e_{1},g_{2}\rangle +e^{i\chi }\sqrt{c}|g_{1},e_{2}\rangle )$$

where *a, b, c*, and *d* are the independent parameters governing the nature of the initial state of the two entangled atoms. They satisfy the relation (a+b+c+d)/3=1, and χ is the initial phase. In fact, the initial states considered in this work are of practical interest and have been already constructed before experimentally in different types of quantum systems. For instance, the Bell state has been created in different systems, such as the trapped ions in the pioneering work of Blatt and Wineland [53] and other works, in particular, that studied the Bell inequality testing in spins in nitrogen-vacancy centers, optical photons, neutral atoms, and superconducting qubits [54,55,56,57,58]. The Werner state was also prepared experimentally via spontaneous parametric conversion and controllable depolarization and decoherence of photons [59,60].

For an open quantum system coupled to a Markovian environment, the system dynamics is represented by the Lindblad master equation [2,61,62]:

(7) $$\dot{\rho }\left(t\right)=-i\left[H,\rho \right]+D_{\rho }\;.$$

where $D_{\rho }$ describes the non-unitary dynamics of the system:

(8) $$D_{\rho }=-\frac{1}{2}\sum_{j=1}^{M}\sum_{k=1}^{N}\left\{\left[L_{k}^{\left(j\right)}\rho ,L_{k}^{(j)†}\right]+\left[L_{k}^{\left(j\right)},\rho L_{k}^{(j)†}\right]\right\}\;,$$

where the *j*th Lindblad operator $L_{k}^{\left(j\right)}$ represents the effect of the considered environment on the system site *k*. Recasting the density operator as a vector in the Liouville space [62], Equation (7) can be rewritten in a matrix form as

(9) $$\vec{\dot{\rho }}\left(t\right)=\left({\hat{L}}^{H}+{\hat{L}}^{D}\right)\vec{\rho }=\hat{L}\vec{\rho }\;,$$

where ${\hat{L}}^{H}$ and ${\hat{L}}^{D}$ are superoperators acting on the vector ρ in the Liouville space, representing the unitary and dephasing (dissipative) processes, respectively. The solution of Equation (9) yields the density matrix at any time *t* as

(10) $$\vec{\rho }\left(t\right)=\sum_{i}A_{i}\;{\vec{\eta }}_{i}\;e^{\lambda_{i}\;t}\;,$$

The coefficient $A_{i}$ is determined by the system’s initial conditions. $\left\{\lambda_{i}\right\}$ is the set of eigenvalues, and $\left\{{\vec{\eta }}_{i}\right\}$ is the set of eigenvectors of the tetrahedral matrix L, which are obtained by exact numerical diagonalization. In our case, the Lindblad operator representing the dephasing environment and acting on the atom *j* is given by $S_{j}^{z}$, where in the pure dephasing case, each atom (qubit) is exposed to an independent dephasing environment, whereas in the second case, the two qubits are exposed to a common correlated dephasing environment.

As a result, $L^{D}$ takes the form

(11) $$\begin{array}{ccc} L^{D} & = & -\sum_{j=1,2}\Gamma_{j}\left(S_{j}^{Z}S_{j}^{Z}\rho +\rho S_{j}^{Z}S_{j}^{Z}-2S_{j}^{Z}\rho S_{j}^{Z}\right)\\ & & -2\Gamma_{0}\left(S_{1}^{Z}S_{2}^{Z}\rho +\rho S_{1}^{Z}S_{2}^{Z}-S_{1}^{Z}\rho S_{2}^{Z}-S_{2}^{Z}\rho S_{1}^{Z}\right). \end{array}$$

where $\Gamma_{j}$ is the dephasing rate of the *j*th atom and $2\Gamma_{0}$ is the correlated dephasing rate. After some calculations, the Liouville operator takes the matrix form:

(12) $$L=\frac{1}{4}\left(\begin{array}{cccccccccccccccc} 0 & 0 & 0 & i\gamma & 0 & 0 & 0 & 0 & 0 & 0 & 0 & 0 & -i\gamma & 0 & 0 & 0\\ 0 & \epsilon_{2}^{-} & i & 0 & 0 & 0 & 0 & 0 & 0 & 0 & 0 & 0 & 0 & -i\gamma & 0 & 0\\ 0 & i & \epsilon_{1}^{-} & 0 & 0 & 0 & 0 & 0 & 0 & 0 & 0 & 0 & 0 & 0 & -i\gamma & 0\\ i\gamma & 0 & 0 & \beta_{1}^{-} & 0 & 0 & 0 & 0 & 0 & 0 & 0 & 0 & 0 & 0 & 0 & -i\gamma \\ 0 & 0 & 0 & 0 & \epsilon_{2}^{+} & 0 & 0 & i\gamma & -i & 0 & 0 & 0 & 0 & 0 & 0 & 0\\ 0 & 0 & 0 & 0 & 0 & 0 & i & 0 & 0 & -i & 0 & 0 & 0 & 0 & 0 & 0\\ 0 & 0 & 0 & 0 & 0 & i & \beta_{1}^{+} & 0 & 0 & 0 & -i & 0 & 0 & 0 & 0 & 0\\ 0 & 0 & 0 & 0 & i\gamma & 0 & 0 & \alpha_{1}^{+} & 0 & 0 & 0 & -i & 0 & 0 & 0 & 0\\ 0 & 0 & 0 & 0 & -i & 0 & 0 & 0 & \epsilon_{1}^{+} & 0 & 0 & i\gamma & 0 & 0 & 0 & 0\\ 0 & 0 & 0 & 0 & 0 & -i & 0 & 0 & 0 & \beta_{2}^{+} & i & 0 & 0 & 0 & 0 & 0\\ 0 & 0 & 0 & 0 & 0 & 0 & -i & 0 & 0 & i & 0 & 0 & 0 & 0 & 0 & 0\\ 0 & 0 & 0 & 0 & 0 & 0 & 0 & -i & i\gamma & 0 & 0 & \alpha_{2}^{+} & 0 & 0 & 0 & 0\\ -i\gamma & 0 & 0 & 0 & 0 & 0 & 0 & 0 & 0 & 0 & 0 & 0 & \beta_{2}^{-} & 0 & 0 & i\gamma \\ 0 & -i\gamma & 0 & 0 & 0 & 0 & 0 & 0 & 0 & 0 & 0 & 0 & 0 & \alpha_{1}^{-} & i & 0\\ 0 & 0 & -i\gamma & 0 & 0 & 0 & 0 & 0 & 0 & 0 & 0 & 0 & 0 & i & \alpha_{2}^{-} & 0\\ 0 & 0 & 0 & -i\gamma & 0 & 0 & 0 & 0 & 0 & 0 & 0 & 0 & i\gamma & 0 & 0 & 0 \end{array}\right),$$

where

(13) $$\begin{array}{cc} \epsilon_{k}^{\pm } & =-4\Gamma_{k}\pm 2i\left(\delta +2\omega_{k}\right)\\ \alpha_{k}^{\pm } & =-4\Gamma_{k}\pm 2i\left(\delta -2\omega_{k}\right)\\ \beta_{1}^{\mp } & =4(\mp 2\Gamma_{0}-\Gamma_{1}-\Gamma_{2}-i\left(\omega_{1}\pm \omega_{2}\right))\\ \beta_{2}^{\pm } & =4(\pm 2\Gamma_{0}-\Gamma_{1}-\Gamma_{2}+i\left(\omega_{1}\mp \omega_{2}\right)) \end{array}$$

and k=1,2.

## 3. Dynamics of Entanglement

A comprehensive view of the system can be gained by investigating the dynamics of the bipartite entanglement that arises naturally between the two atoms and the atomic population inversion starting from different initial states of particular interest. In this section, we implemented our solution to study the dynamics of the entanglement of the system.

The entanglement can be quantified via the aid of the concurrence C(ρ) as proposed by Wootters [63]. It can be calculated from

(14) $$\begin{array}{c} C\left(\rho \right)=\max\;\left[0,\varepsilon_{1}-\varepsilon_{2}-\varepsilon_{3}-\varepsilon_{4}\right], \end{array}$$

where $\varepsilon_{i}$ in decreasing order are the square roots of the four eigenvalues of the non-Hermitian matrix:

(15) $$\begin{array}{c} R\equiv \rho \tilde{\rho }, \end{array}$$

where $\tilde{\rho }$ is the spin flipped state defined as

(16) $$\begin{array}{c} \tilde{\rho }=\left({\hat{\sigma }}_{y}⊗{\hat{\sigma }}_{y}\right)\rho^{*}\left({\hat{\sigma }}_{y}⊗{\hat{\sigma }}_{y}\right), \end{array}$$

Here, $\rho^{*}$ is the complex conjugate of ρ and ${\hat{\sigma }}_{y}$ is the Pauli spin matrix in the *y* direction. In general, it is known that C(ρ) goes from 0 for a separable disentangled state to 1 for a maximally entangled state. Since the main goal of our work is to investigate the impact of the asymmetry of the two qubits on the system dynamics and entanglement properties, the asymmetry is reflected in two aspects, the difference in the qubit transition frequencies (energy gaps) and their coupling strengths to the environment. Therefore, in our model, which is generic, we assumed one of the two frequencies, $\omega_{1}$, is equal to one, while the other one is weighted in terms of the first. Consequently, we can study the different scenarios at different ratios of $\omega_{1}$ and $\omega_{2}$. Furthermore, since we are working in a unit system where ℏ=1, all the frequencies and coupling strengths are expressed in units of $\omega_{1}$ and the time *t* in units of $\omega_{1}^{-1}$. When our model is applied to one of the relevant physical systems, we can assign a numerical value to the frequencies. For instance, in a system such as the trapped ions, the energy gap, and therefore $\hbar \omega_{1}$, is around $10^{14}$ Hz, whereas in the superconducting qubits, it is a few GHz [64].

### 3.1. Disentangled State

**Figure 1:**
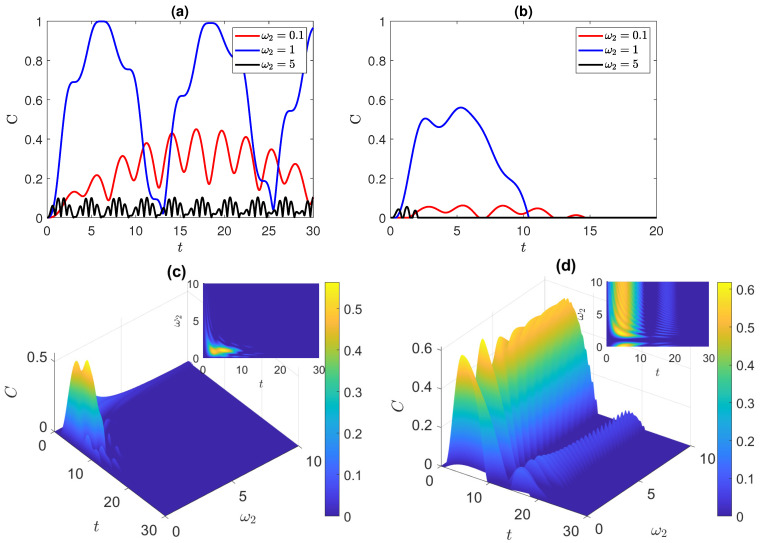
Dynamics of entanglement starting from the disentangled state ${|\psi \left(0\right)\rangle }_{D}=(|e_{1},e_{2}\rangle +|e_{1},g_{2}\rangle +|g_{1},e_{2}\rangle +|g_{1},g_{2}\rangle )/2$, where $\omega_{1}=1$ and varying $\omega_{2}$ in the: (**a**) Ising system in the absence of environments; (**b**,**c**) Ising system in the presence of uncorrelated dephasing environment; (**d**) XXX system in the presence of uncorrelated dephasing environment. The dephasing parameters are set to $\Gamma_{1}=10$$\Gamma_{2}=0.1$.

We start by considering the time evolution of the system, at different degrees of anisotropy, starting from the disentangled initial state ${|\psi \left(0\right)\rangle }_{D}=(|e_{1},e_{2}\rangle +|e_{1},g_{2}\rangle +|g_{1},e_{2}\rangle +|g_{1},g_{2}\rangle )/2$. The system is coupled to a pure dephasing environment characterized by dephasing rates $\Gamma_{1}=0.1$ and $\Gamma_{2}=0.01$. For the rest of this paper, we use these values of the dephasing rates unless otherwise stated explicitly. The dynamics of the entanglement between the two qubits for different values of $\omega_{2}$, while keeping $\omega_{1}=1$, are depicted in Figure 1a,b. Figure 1a shows the dynamics for a closed system in absence of a dephasing environment. Each $\omega_{2}$ value exhibits a distinct, continuous, and irregular oscillatory behavior. Notably, maximum entanglement is achieved when $\omega_{2}\neq \omega_{1}$, with varying amplitudes among the peaks. Further examination of the entanglement evolution reveals a quasi-periodic pattern of oscillations. In cases where $\omega_{2}\neq \omega_{1}$, both the entanglement amplitude and the shape of the oscillations are disturbed. Specifically, when $\omega_{2}=0.1$, the entanglement is more pronounced compared to the case when $\omega_{2}=5$. It is worth noting that, while the entanglement begins from zero, once it emerges, it persists and never vanishes. Meanwhile, as can be noticed from Figure 1b, in the presence of a pure dephasing environment, the entanglement ends up vanishing after a period of time that varies depending on the difference between the two frequencies. The highest peak of entanglement is again observed when $\omega_{2}=\omega_{1}$, but here with smaller entanglement content. For $\omega_{2}=0.1$, three peaks that are intermediated by ESD appear, and finite disentanglement occurs just after the third peak. Though when $\omega_{2}=2$, the entanglement persists for a longer duration compared to the dynamics of the other frequencies, initially, the ESB is delayed and the peaks have small amplitudes that are comparable to those of $\omega_{2}=0.1$. Moreover, ESD occurs twice, with the amplitude of the final revived peak being notably smaller. Figure 1c displays the time evolution of entanglement versus the frequency of the second qubit in the completely anisotropic (Ising) system. In this state, the two atoms initially possess zero entanglements, then become entangled for a finite time before becoming disentangled again. We observe that, when the frequencies of the two qubits are close to each other, with a difference of less than one atomic transition level, the entanglement reaches a maximum value of approximately 0.55, and the entanglement persists for a longer duration. As the second atom’s frequency $\omega_{2}$ deviates further away from the first atom’s frequency $\omega_{1}$, the entanglement oscillations become shorter and experience more-frequent occurrences of entanglement sudden death (ESD) and revivals, ultimately leading to faster disentanglement.

The dynamics of an Isotropic (XXX) system shows a distinct behavior from the Ising system, as shown in Figure 1d. A preliminary overview of the 3D plot shows that, as $\omega_{2}$ increases, the period of the initial disentanglement between the two atoms decreases, while the maxima of entanglement become smaller. Furthermore, the plot shows that, when the frequencies of the two atoms are close to each other, the atoms maintain their disentanglement status. However, when the atoms exhibit asymmetry, particularly when the frequency of the second atom is roughly double that of the first atom, the system successfully establishes entanglement between the atoms, reaching a maximum entanglement value of 0.6, which surpasses the value attained in the Ising model. Furthermore, the entanglement in the XXX system appears to persist for a longer duration compared to the Ising system, with disentanglement occurring around t = 20, whereas in the Ising system, the longest period of entanglement lasts until t = 15.

**Figure 2:**
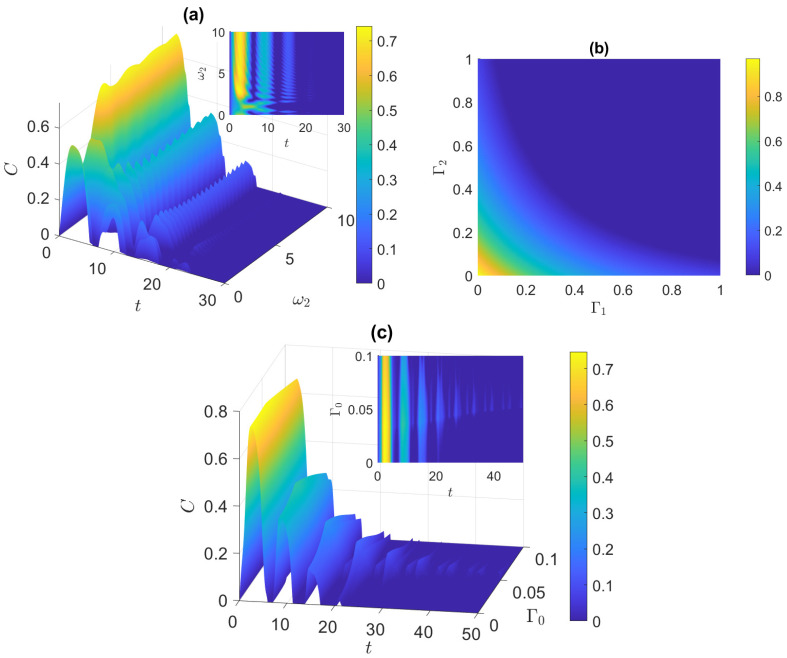
Starting from the disentangled state ${|\psi \left(0\right)\rangle }_{D}=(|e_{1},e_{2}\rangle +|e_{1},g_{2}\rangle +|g_{1},e_{2}\rangle +|g_{1},g_{2}\rangle )/2$, in the XYZ system: (**a**) dynamics of entanglement vs. $\omega_{2}$, at $\omega_{1}=1$; (**b**) entanglement vs. $\Gamma_{1}$ and $\Gamma_{2}$, at t=5/2 and $\omega_{2}=3$; (**c**) dynamics of entanglement vs. $\Gamma_{0}$, at $\omega_{2}=3$.

In Figure 2, we continue our investigation into the dynamics of the system starting from the initially disentangled state. Here, we considered a system with a partial degree of anisotropy (XYZ system). In Figure 2a, which examines the entanglement evolution for $0<\omega_{2}<10$, we observe that the initially disentangled atoms gain entanglement regardless of whether the atoms are symmetric or asymmetric. We note that, when the atoms are symmetric or close to symmetry, the entanglement of the atoms occurs once with a peak that reaches a certain height before decaying, as shown in the inset. However, when the atoms are asymmetric with the value of $\omega_{2}$ above 2, the atoms experience ESD at least three times; the ESD period becomes longer after each revival, and the amplitude of the peaks decreases. It is noteworthy that, for this XYZ system, the entanglement reaches a maximum amplitude of approximately 0.7 when $\omega_{2}=3$. This maximum value is higher than what was observed in both the Ising and XXX models. Next, we study in Figure 2b the effect of varying the independent dephasing rates $\Gamma_{1}$ and $\Gamma_{2}$. Since the highest peak of entanglement was observed for $\omega_{2}=3$ at t=5/2, we investigated the state of the system at that instance. When the dephasing rates are very low, the entanglement reaches a value of 0.96, indicating that the atoms are almost completely entangled. As the dephasing rates increase, the entanglement decreases. In fact, a similar effect was observed in the XXX and Ising models. It was also found that the variation of the pure dephasing rates depend on the anisotropy of the system, where in the XYZ system, increasing $\Gamma_{1}$ increases the decay rate slower than increasing $\Gamma_{2}$, while in the Ising system, it occurs the other way around. On the other hand, in the isotropic XXX system, the variation of $\Gamma_{1}$ and $\Gamma_{2}$ exhibits a symmetric dephasing rate, such that increasing either one of them increases the dephasing rate the same amount. In Figure 2c, we examine the time evolution of entanglement as a function of the coupled dephasing rate $\Gamma_{0}$ when $\omega_{2}=3$. At a given $\Gamma_{0}$, the entanglement exhibits an oscillatory behavior with a collapse revival pattern. Interestingly, the entanglement appears to be enhanced as we increase $\Gamma_{0}$, in particular when $0.05<\Gamma_{0}<0.07$, where the enhancement reaches its maximum. New collapse and revival peaks are created in this range, leading to a delay in the disentanglement of the two atoms. However, increasing $\Gamma_{0}$ further has a detrimental effect on the entanglement. The XXX and Ising models exhibit a comparable effect as the one depicted in Figure 2a,b. However, for the variation of entanglement with the independent dephasing rates, the rate at which $\Gamma_{i}$ accelerates the dephasing was found to be faster in the other two models compared with the XYZ one.

### 3.2. Correlated Bell State

**Figure 3:**
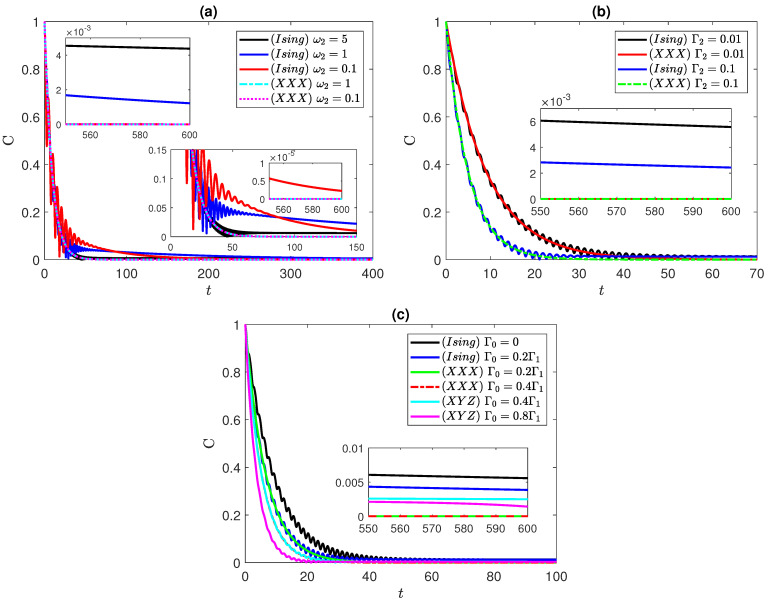
Dynamics of entanglement starting from the correlated Bell state, $\psi_{Bc}=(|e_{1}\rangle |e_{2}\rangle +|g_{1}\rangle |g_{2}\rangle )/\sqrt{2}$, in the Ising, XXX, and XYZ systems at different values of: (**a**) $\omega_{2}$ at $\Gamma_{1}=0.1$, and $\Gamma_{2}=0.01$; (**b**) $\Gamma_{2}$ at $\Gamma_{1}=0.1$ and $\Gamma_{0}=0$; (**c**) $\Gamma_{0}$ at $\Gamma_{1}=0.1$, and $\Gamma_{2}=0.01$. $\omega_{1}=1$ in all panels.

Figure 3 shows the dynamics of entanglement starting from the maximally entangled correlated Bell state $\psi_{Bc}=(|e_{1}\rangle |e_{2}\rangle +|g_{1}\rangle |g_{2}\rangle )/\sqrt{2}$.

In the Ising system case, depicted in Figure 3a, the entanglement starts at a maximum value and gradually decays until completely vanishing, where at $\omega_{2}=0.1$ and 1, we note an oscillatory behavior. A very similar effect was observed in the XYZ system, which we do not show here to avoid redundancy. In contrast, we found that the entanglement in the XXX system exhibits no oscillations and is independent of the frequency of the second atom, as depicted in the figure for $\omega_{2}=0.1$ and 1, where the curves coincide with each other. Interestingly, the impact of varying the pure dephasing rate on the entanglement dynamics was found to be similar across the Ising, XXX, and XYZ systems, irrespective of the anisotropy variation; again, we do not plot the XYZ system dynamics due to the close similarity to the Ising case. It is remarkable that the Ising system sustains its entanglement for a long period of time before vanishing, and as can be noticed in the inner insets in Figure 3a, that period of time increase with the frequency $\omega_{2}$, while the entanglement decays very slowly with time. This can be an advantage for quantum information processing in such systems where the entanglement persists for a long period of time despite the dephasing effect. We show the entanglement behavior, in Figure 3b, in the Ising and XXX systems, where $\Gamma_{1}$ is fixed to 0.1, while $\Gamma_{2}$ is varying. When $\Gamma_{2}=0.01$, the dynamics of both systems follow the same trace. However, since $\omega_{2}=3$, the Ising system exhibits oscillatory behavior in its dynamics for which, at this value of $\omega_{2}$, the oscillations of the Ising system remains clearly observable. Increasing $\Gamma_{2}$ to 0.1 results in a damping effect on the entanglement and an accelerated rate of dephasing, leading to faster disentanglement of the atoms. Nevertheless, the dynamics of both systems ultimately decay at the same rate. It is worth mentioning that the oscillations in the Ising system slightly delay the disentanglement process as the entanglement approaches low values, whereas for the XXX system with $\Gamma_{2}=0.1$, the entanglement vanishes faster compared to the Ising system with the same $\Gamma_{2}$ value. Again, one can notice in the insets of Figure 3b how the entanglement of the Ising system persists for a long period of time before vanishing, where the period increases as the uncorrelated dephasing strength decreases, whereas that of the XXX system vanishes much earlier at the same values of the dephasing strengths. The impact of varying the correlated dephasing rate is investigated in Figure 3c. Unlike the disentangled state, this parameter induces only a damping effect on the entanglement. The change in the dephasing rate across all systems demonstrates a nearly identical behavior as $\Gamma_{0}$ increases. This is illustrated by first inspecting the change in the dephasing rate for the Ising system by varying $\Gamma_{0}$ from 0 to $0.2\Gamma_{1}$. Subsequently, observing the effect on the XXX system at $\Gamma_{0}=0.2\Gamma_{1}$, we observe alignment between the Ising and XXX system lines. It is important to note that the XXX system does not exhibit oscillations in the entanglement dynamics as discussed in Figure 3a. Therefore, at low entanglement levels, oscillations arising from the asymmetric XY interaction lead to a delay in disentanglement. Additionally, we further explore the change by increasing $\Gamma_{0}$ from 0.4 to 0.8. As expected, the dynamics of the XXX and XYZ systems align with each other at $\Gamma_{0}=0.4\Gamma_{1}$. The behavior of the entanglement in the insets of Figure 3c shows that the entanglement in both of Ising system and XYZ system, with partial and complete anisotropy, persists for a long period of time, which is higher in the Ising system and decreases as the correlated dephasing strength decreases. On the other hand, the entanglement in the isotropic (XXX) system vanishes very early, which indicates that a stronger spin–spin coupling in one direction resits the dephasing impact efficiently.

### 3.3. Anti-Correlated Bell State

**Figure 4:**
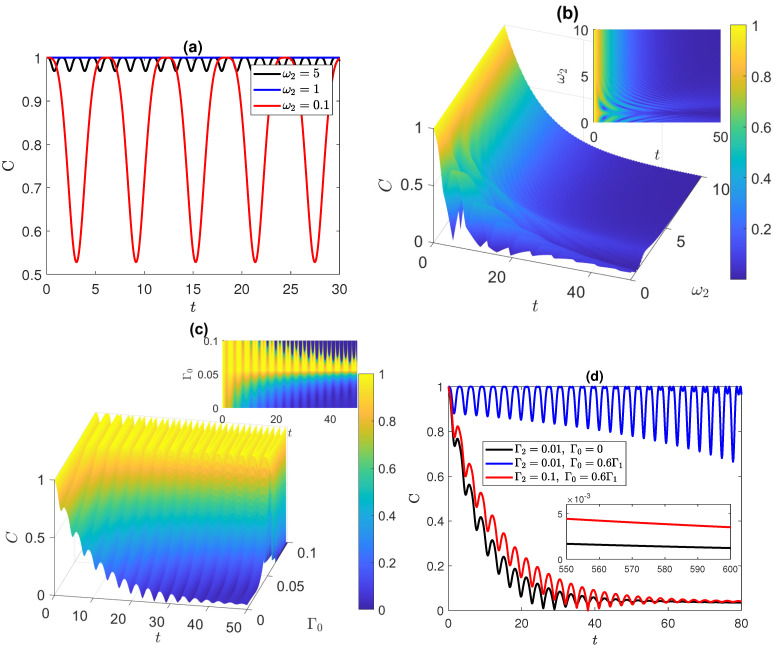
Dynamics of entanglement starting from the anti-correlated Bell state, ${|\psi \left(0\right)\rangle }_{Ba}=(|e_{1},g_{2}\rangle +|g_{1},e_{2}\rangle )/\sqrt{2}$, in the XXX and XYZ systems: (**a**) at different values of $\omega_{2}$ in the absence of environments; (**b**) vs. $\omega_{2}$ at $\Gamma_{1}=0.1$, $\Gamma_{2}=0.01$, and $\Gamma_{0}=0$; (**c**) vs. $\Gamma_{0}$ at $\Gamma_{1}=0.1$ and $\Gamma_{2}=0.01$; (**d**) at different values of $\Gamma_{0}$ and $\Gamma_{2}$, at $\Gamma_{1}=0.1$ and $\omega_{2}=3$. $\omega_{1}=1$ in all panels.

Another maximally entangled initial state is examined in Figure 4, namely the anti-correlated Bell state (ACBS) ${|\psi \left(0\right)\rangle }_{Ba}=(|e_{1},g_{2}\rangle +|g_{1},e_{2}\rangle )/\sqrt{2}$. Surprisingly, all three systems exhibit the same dynamics shown in Figure 4. As illustrated in Figure 4a, it is evident that in a closed system, when $\omega_{2}=\omega_{1}$, the entanglement remains constant at a value of 1. However, when $\omega_{2}\neq \omega_{1}$, the dynamics experience an oscillatory behavior with varying periods determined by $\omega_{2}$. For example, when $\omega_{2}=0.1$, the period of the oscillations is longer compared to the case when $\omega_{2}=5$, where smaller oscillations are observed, maintaining the entanglement close to 1 over time. When introducing the pure dephasing environment in Figure 4b while varying $\omega_{2}$, we observe that the entanglement decays faster for symmetric atoms (with $\omega_{2}=\omega_{1}$=1) compared to the asymmetric atoms case. Remarkably, when $\omega_{2}=2$, the entanglement between the atoms is preserved for a longer duration, while increasing $\omega_{2}$ further leads to accelerated decay of entanglement. Investigating the effect of the independent dephasing environments on the ACBS case shows that increasing either $\Gamma_{1}$ or $\Gamma_{2}$ results in a symmetric impact on the entanglement dynamics, similar to the findings for the disentangled and correlated Bell states.Varying $\Gamma_{0}$ in Figure 4c reveals an intriguing observation. Although we retrieved an evolution that is similar to the initially disentangled state case, where an increase in $\Gamma_{0}$ enhances the entanglement and delays the disentanglement, particularly when $\Gamma_{0}$ is within the range of $0.5\Gamma_{1}$ to $0.7\Gamma_{1}$, for this case, the enhancement is more pronounced compared to the previous case, with the entanglement being maintained at maximum values for an extended period of oscillations. To further explore the dynamics, we examine the combined effect of varying both $\Gamma_{2}$ and $\Gamma_{0}$ in Figure 4d.

Increasing $\Gamma_{0}$ from 0 to $0.6\Gamma_{1}$ leads to a significant enhancement in entanglement, resulting in a prolonged period of maximum entanglement. Nevertheless, the minima of the oscillations decrease with time, evoking a less-stable entanglement that leads eventually to disentanglement. On the other hand, increasing $\Gamma_{2}$ from 0.01 to 0.1 portrays the dephasing effect of the independent environment. The behavior of the entanglement in the insets of Figure 4d demonstrates that the entanglement in both of the XXX and XYZ system, starting form the state ${|\psi \left(0\right)\rangle }_{Ba}$, persists for a long period of time against the dephasing effects, and surprisingly, the higher dephasing values $\Gamma_{2}=0.1$ and $\Gamma_{0}=0.6\Gamma_{1}$ lead to longer periods of time compared with $\Gamma_{2}=0.01$ and $\Gamma_{0}=0$.

### 3.4. W and Werner States

**Figure 5:**
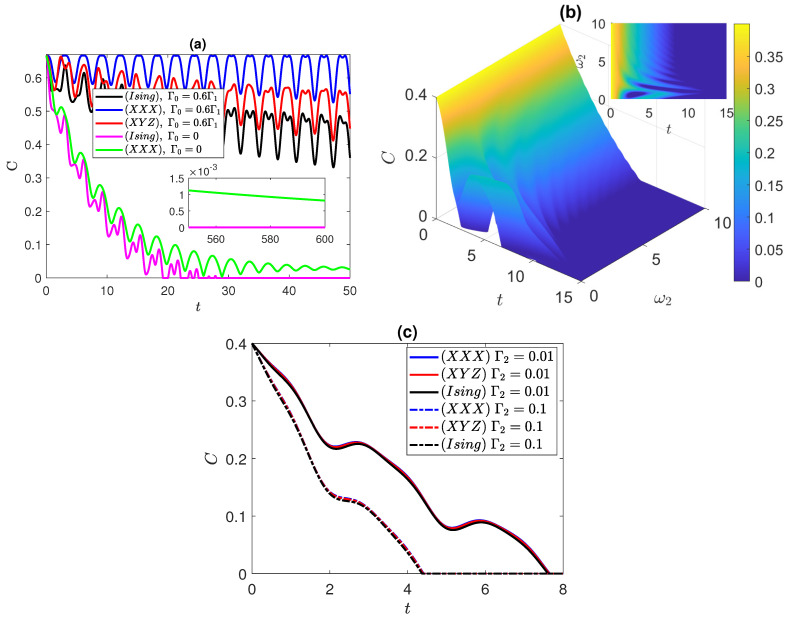
Dynamics of entanglement starting from (**a**) the W-state, ${|\psi \left(0\right)\rangle }_{W}=(|e_{1},g_{2}\rangle +|g_{1},e_{2}\rangle +|g_{1},g_{2}\rangle )/\sqrt{3}$, in the Ising, XXX, and XYZ systems at $\omega_{1}=1$, $\omega_{2}=3$, $\Gamma_{1}=0.1$, $\Gamma_{2}=0.01$, and different values of $\Gamma_{0}$; (**b**) Werner state in the XYZ system, vs. $\omega_{2}$, at $\omega_{1}=1$, $\Gamma_{1}=0.1$, $\Gamma_{2}=0.01$, and $\Gamma_{0}=0$; (**c**) Werner state in the three systems at $\omega_{1}=1$, $\omega_{2}=3$, $\Gamma_{1}=0.1$, $\Gamma_{0}=0$, and different values of $\Gamma_{2}$.

In Figure 5, we examine the initial partial entangled state and mixed state. Figure 5a presents a system initialized in the W-state ${|\psi \left(0\right)\rangle }_{W}=(|e_{1},g_{2}\rangle +|g_{1},e_{2}\rangle +|g_{1},g_{2}\rangle )/\sqrt{3}$. We note that the behavior of the XXX system, as $\Gamma_{0}$ varies, resembles the behavior of the ACBS in which the entanglement oscillations exhibit multiple peaks with a maximum amplitude when $0.5\;\Gamma_{1}<\Gamma_{0}<0.7\;\Gamma_{1}$. In the Ising and XYZ models, the peaks gradually decrease with each oscillation, and the decay rate is faster in the Ising system compared to the XYZ one. On the other hand, the variation of $\Gamma_{1}$ and $\Gamma_{2}$ exhibits a symmetric dephasing rate in the isotropic XXX system starting from the W-state, whereas in the Ising and XYZ systems, increasing $\Gamma_{1}$ increases the decay rate slower than in the case of increasing $\Gamma_{2}$. When investigating the effect of varying $\omega_{2}$ versus *t* in the W-state, we observe that the entanglement dynamics of the XXX system share a similar two-dimensional projection with the ACBS, albeit with a faster decay due to the W-state being a partially entangled state with less initial entanglement content. The dynamics of the Ising and XYZ systems display more oscillations that experience ESD several times, leading to a faster decay, with the decay rate being higher in the Ising model than in the XYZ model.

The inset of Figure 5a illustrates, starting from the state ${|\psi \left(0\right)\rangle }_{W}$, in contrast to what we have observed before, that the entanglement in the Ising system vanishes very early, while that of the XXX system persists for a long period of time before vanishing in the absence of correlated dephasing. The final state under consideration is the Werner state, with the initial parameters taken as a=0.2, b=1, c=1, d=1−a, and χ=π/4. Varying $\omega_{2}$ in Figure 5b, one can notice that, when the value of $\omega_{2}$ is close to $\omega_{1}$, the entanglement decreases and experiences ESD, which is followed by a revival peak. This peak delays the disentanglement of the atoms as shown in the figure, while for higher values of $\omega_{2}$, the disentanglement occurs earlier. For the effect of $\Gamma_{0}$, we obtained dynamics that follow the same pattern as the ACBS, except that the maximum of the entanglement is 0.4, not 1. This pattern applies to all the distinct systems, with fine variations in the entanglement evolution among them. In Figure 5c, we demonstrate the matching effect of the independent dephasing environment over the different systems. First, we display the dynamics at $\Gamma_{2}=0.01$ in the three systems, which yields distinct, yet highly similar curves for each system. Then, by increasing $\Gamma_{2}$ to 0.1, we obtain three additional curves that undergo a similar behavior of the decaying rate.

## 4. Conclusions

We considered a system of two interacting two-level non-identical atoms (qubits) coupled to pure (uncorrelated) and correlated dephasing environments. We studied the system dynamics starting from different initial states that vary in the degree of purity and entanglement content. We tested the impact of the asymmetry of the two-qubit system on the entanglement dynamics and asymptotic behavior. It was found that the differences in the two qubits’ frequencies and coupling strengths to the uncorrelated dephasing environment vary considerably depending on the initial state and degree of anisotropy of interaction between the two qubits. Starting from certain initial states, such as the disentangled and Werner states, increasing the difference between the frequencies of the two qubits leads to higher entanglement decay rates, which are reduced as the degree of anisotropy increases in the initial disentangled state case. In contrast, starting from an anti-correlated Bell state, equal frequencies would lead to higher entanglement decay rates at different degrees of anisotropy. In general, the deviation between the coupling strengths of the two qubits to the uncorrelated dephasing environment yields a higher decay of entanglement, though its rate varies with the degree of anisotropy and the initial state type. The coupling of the two qubits to the correlated dephasing environment was found to be useful, enhancing the entanglement, within a certain range of values of the coupling strength, for specific initial states, such as the disentangled, Werner, and anti-correlated Bell states, whereas it is devastating in the case of other initial states such as the correlated Bell state.

## Data Availability

The data presented in this study is contained within the article.
